# The Dental Association of Western New York

**Published:** 1867-11

**Authors:** 


### THE DENTAL ASSOCIATION OF WESTERN NEW YORK.

THIS Body held its regular semi-annual meeting at Lockport, on the first Tuesday of October. As is usual with this Association, this meeting was a good one. It is composed of men who are working earnestly for the elevation and prosperity of our profession. The subject of legal protection for the profession and the people of the State of New York from Dental empiricism was considered at some length, and a committee was appointed to take action in the matter, with special reference to laying it before the Legislature, and we sincerely hope their efforts may be crowned with success.

The time is rapidly approaching, if it is not already at hand, when our Associations will be constrained to take up, consider and act upon other subjects than merely a routine of business, and the discussion of modes of practice and subjects immediately connected with it. While we fully recognize that our profession requires all that has been done in this direction, and far more, yet it is becoming more and more apparent that there are other matters bearing strongly upon the profession, that cannot be much longer overlooked or forgotten; and this subject of legal recognition we regard as one of them. And again, the subject of professional education is one that must be taken hold of by the profession with far more earnestness than hitherto; something in this direction has been done by a few, and a very few of our associations. Why is it that our colleges are not far more efficient than they are? Simply because the profession has not demanded it; and the profession can only make its voice heard forcibly through its associations.

We have for a long time felt that the members of our profession do not recognize and appreciate the power there is in associated effort. We know that many place altogether too low an estimate on this instrumentality. Dental Associations have thus far, for the most part, been too feebly organized; and we would suggest that all of our more important societies become incorporated,—possess a legal existence; and that members value them more than heretofore, and let not trifling things keep them from the meetings; and when present, always be prepared each to perform his full share of the work. T.

